# Co-Infection of Swine with Porcine Circovirus Type 2 and Other Swine Viruses

**DOI:** 10.3390/v11020185

**Published:** 2019-02-21

**Authors:** Ting Ouyang, Xinwei Zhang, Xiaohua Liu, Linzhu Ren

**Affiliations:** 1 Jilin Provincial Key Laboratory of Animal Embryo Engineering, College of Animal Sciences, Jilin University, 5333 Xi’an Road, Changchun 130062, China; ouyt17@mails.jlu.edu.cn (T.O.); xwzhang17@mails.jlu.edu.cn (X.Z.); lxh18@mails.jlu.edu.cn (X.L.); 2 College of Life Sciences, Shandong Normal University, Jinan 250014, China

**Keywords:** porcine circovirus 2 (PCV2), co-infection, swine, virus

## Abstract

Porcine circovirus 2 (PCV2) is the etiological agent that causes porcine circovirus diseases and porcine circovirus-associated diseases (PCVD/PCVAD), which are present in every major swine-producing country in the world. PCV2 infections may downregulate the host immune system and enhance the infection and replication of other pathogens. However, the exact mechanisms of PCVD/PCVAD are currently unknown. To date, many studies have reported that several cofactors, such as other swine viruses or bacteria, vaccination failure, and stress or crowding, in combination with PCV2, lead to PCVD/PCVAD. Among these cofactors, co-infection of PCV2 with other viruses, such as porcine reproductive and respiratory syndrome virus, porcine parvovirus, swine influenza virus and classical swine fever virus have been widely studied for decades. In this review, we focus on the current state of knowledge regarding swine co-infection with different PCV2 genotypes or strains, as well as with PCV2 and other swine viruses.

## 1. Introduction

The porcine circovirus (PCV) belongs to the family *Circoviridae* and contains a single-stranded circular DNA genome [1,2,3]. There are three types of PCV: porcine circovirus type 1 (PCV1), porcine circovirus type 2 (PCV2) and porcine circovirus 3 (PCV3). During the past few decades, PCV2 has been widely studied and is considered to be the main pathogen responsible for porcine circovirus diseases and porcine circovirus-associated diseases (PCVD/PCVAD), which are characterized as clinical or subclinical PCV2 infections among pigs [2,4]. The most representative symptoms of the diseases include porcine dermatitis and nephropathy syndrome (PDNS), which mainly occurs during the growing or finishing stage of pigs; postweaning multisystemic wasting syndrome (PMWS), which affects nursery and growing pigs; and porcine respiratory disease complex (PRDC), which usually occurs in pigs 14–20 weeks of age [1,2,3,4,5].

To date, the exact mechanisms of PCVD/PCVAD are currently unknown [2]. However, many studies have reported co-infection with other swine pathogens, such as porcine reproductive and respiratory syndrome virus, porcine parvovirus, swine influenza virus, *Mycoplasma hyopneumoniae*, and *Salmonella* spp., are important cofactors that may enhance PCV2 infection and the severity of PCVD/PDVAD [2,6,7,8,9,10,11,12,13,14,15]. Furthermore, vaccination failure, stress or crowding together with PCV2-infected animals also cause PCVD/PCVAD [5,16,17,18,19,20]. As co-infections with viruses are frequently detected in domestic pigs and wild boars, we discuss co-infections of pigs with PCV2 and other swine viruses in this review. Furthermore, co-infections of different PCV2 strains, which cause recombination and genomic shifts in recent years, are also reviewed.

## 2. Co-Infection with Different Porcine Circovirus 2 (PCV2) Strains

PCV2 is divided into five genotypes according to the Cap gene sequence: PCV2a, 2b, 2c, 2d, and 2e [2,21]. Moreover, the PCV2b genotype is classified into three clusters, 1A to 1C, and the PCV2a genotype is subdivided into five clusters, 2A to 2F [2,22,23,24]. Recently, a retrospective study of PCV2 infection between 1996 and 1999 in China revealed a novel genotype PCV2f which shared lower sequence identity with the other known genotypes [25]. Since the discovery of PCV2 in the late 1990s, the virus has continued to evolve, and two major genotype shifts have been observed. The first genotype shift in PCV2 was from PCV2a to PCV2b in 2004/2005 [26,27]. Since 2012, the predominant PCV2b has been gradually replaced by the PCV2d genotype in North America, China, South Korea and Uruguay [22,26,27,28,29,30]. Besides, PCV2F becomes the predominant genotype in the PCV2a cluster in China [22].

It has been reported that concurrent infections with different PCV2 genotypes have been detected in the same pig, resulting in inter- and intra-genotype recombination [13,31,32,33]. One hundred and eighteen PCV2-positive DNA samples isolated from diseased pigs were analyzed using a modified differential polymerase chain reaction (PCR) assay, and the results indicated that the coexistence rates of PCV2 genotypes were 32.2% (38/118) in sick pigs [13]. The sequencing results of 38 co-infected samples showed that the coexisting genotypes were PCV2a-PCV2b (12/38), PCV2a-PCV2d (15/38) and PCV2e-PCV2d (11/38) [13]. One group reported that the recombination rate of the PCV2 isolates was 27.7% (17/54) in the samples collected from 2006 to 2016 in China [34], and the recombination mainly occurred in the ORF1 gene of PCV2 [34,35]. Furthermore, co-infections with different PCV2 genotypes may cause more serious disease. In cells infected with replicating viruses, both PCV2a and PCV2b genotypes were equally present [31]. Further studies have demonstrated that pigs with dually heterologous inoculation or naturally infected with multiple PCV2 genotypes or strains displayed more severe lesions [36,37]. These results suggest that the coexistence of different strains of PCV2 might contribute to the development of more severe clinical symptoms in pigs and more recombination events between strains in the field [13,35,36,37]. Therefore, more studies need to focus on analyzing the recombination trends of PCV2 strains, which may provide a better strategy for vaccine development and vaccination strategy.

## 3. Co-Infection of PCV2 with Other Swine Viruses

### 3.1. Porcine Circovirus 3 (PCV3)

Porcine circovirus 3 (PCV3) is a newly emerging virus belonging to the family *Circoviridae,* and it has a circular 2000 bp ssDNA genome that mainly encodes replicase and capsid proteins [38,39,40]. However, the sequence identity of PCV3 to PCV1 and PCV2 was only 31–48% on the amino acid level, respectively [41], indicating that PCV3 is distinct from PCV1 and PCV2. PCV3 was divided into three major clades (PCV3a, PCV3b and PCV3c) based on complete coding sequences [32,42]. Recently, other groups have found that PCV3 has a close relationship to bat circovirus [32,38,43]. Thus, it was hypothesized that PCV3 may have evolved from bats and then gradually adapted to infect pigs and other animals [43], which might pose a potential risk to public health due to its close relationship with bat circovirus [32,43].

PCV3 can be detected in multiple tissues with different positive rates in healthy and diseased pigs [40,44], and the positive rate of PCV3 has increased gradually in sows and stillborns [44,45]. Surprisingly, the infection rate of PCV3 in wild boars was similar or higher than that of domestic pigs, with the infection rates of 33% to 42.66%, indicating that the wild boar is a potential reservoir for PCV3 [40]. PCV3 has been associated with cardiac and multisystemic inflammation, PRDC, PDNS and reproductive failure [38,39,40,46], most of which are similar to that of PCVD/PCVAD caused by PCV2. Notably, PCV3-positive clinical samples were frequently found to be co-infected with PCV2, as the co-infection rates were 27.6–39.39% and 19.14% in pig farms and slaughter houses, respectively [46,47,48,49,50,51], suggesting mixed infection of PCV2 and PCV3 are common in pig populations. However, to date, isolation and propagation of PCV3 was unsuccessful in vitro [52], which hindered the study of the pathogenesis of PCV3. Both PCV2 and PCV3 mainly infect macrophages in lymphoid tissues [39,53], and therefore, monitoring the prevalence and genetic characteristics as well as co-infection status of PCV2 and PCV3 in clinical samples is an important task for future work. Moreover, PCV2 infection causes immunosuppression in pigs, which provides vital conditions for other pathogens to infect. Two amino acid mutations (A24V and R27K) on the PCV3 Cap protein were supposed to be related to immune escape under immune pressure from the host [44]. Thus, more studies also should focus on the host immune response in the PCV2 and PCV3 co-infected pig.

### 3.2. Porcine Reproductive and Respiratory Syndrome Virus

Porcine reproductive and respiratory syndrome virus (PRRSV) is the etiologic agent that causes porcine reproductive and respiratory syndrome (PRRS), which has been acknowledged as one of the most economically devastating diseases in the swine industry [54,55]. Typical clinical symptoms of PRRS are characterized as blue-ear in the infected pigs, with mild to severe respiratory disease in newborn/weaned piglets and growing pigs, as well as reproductive failure in pregnant pigs [55]. Furthermore, highly pathogenic PRRSV (HP PRRSV) strains are also associated with high fever, respiratory and reproductive failure, pulmonary lesions, and abnormal host immune response [55,56,57].

The co-infection of swine with PRRSV and PCV2 is common in clinical conditions and contributes to a range of polymicrobial disease syndromes [11,29,58]. The co-infection rate of PRRSV and PCV2 has been reported as 42% and up to 85.4% in lungs with proliferative and necrotizing pneumonia lesions in postweaned pigs [59]. A Chinese group reported a co-infection rate of PRRSV and PCV2 of 52.4% in 103 clinical specimens collected from the swine farms of Guizhou province, China [60]. Furthermore, PRRSV- and PCV2-naturally co-infected boars have been found [61].

Both PCV2 and PRRSV can target host immune cells and impair host defenses, resulting in increased susceptibility to infections by primary and secondary pathogens that can affect growth and performance as well as increase morbidity and mortality [58]. Harms and colleagues found that PCV2 and PRRSV co-inoculated pigs had severe dyspnea, lethargy, and occasional icterus by 10 days post-inoculation (dpi) and were dead by 20 dpi, while PCV2-infected pigs developed lethargy, sporadic icterus and exudative epidermitis with mortality rate of 26%, and PRRSV-inoculated pigs only showed dyspnea and mild lethargy [62]. Moreover, PCV2 and PRRSV co-inoculated pigs also had severe proliferative interstitial pneumonia and hepatic lesions, whereas PRRSV-inoculated pigs only had moderate proliferative interstitial pneumonia without bronchiolar or hepatic lesions or lymphoid depletion [62]. Another study demonstrated that replications of PRRSV and PCV2 were enhanced and that more severe clinical signs and lesions were observed in piglets infected with HP-PRRSV followed by PCV2 infection [63]. During the co-infection, PRRSV influences the infection dynamics of PCV2 subtypes PCV2a and PCV2b by lengthening PCV2 viremia and shedding in vivo [64], while PCV2 infection increases the rate of amino acid mutations of PRRSV during serial passages in pigs [65]. In addition, the mutation rates in ORF5 and ORF6 of PRRSV were significantly higher in concurrently PRRSV/PCV2b co-infected pigs than that of pigs infected with PRRSV only, while a significantly higher mutation rate in ORF7 of PRRSV was detected in the PRRSV/PCV2a co-infected pigs [65].

Moreover, it has been reported that the co-infection of pigs with PRRSV and PCV2 results in an increased expression profile of IL-1β and TLR2, 4 and 8, and it has a negatively synergistic effect on the mRNA expression of TLRs 3, 7 and 9, A20, Bcl-3, IRAK-M, MKP-1, SARM1 and SIGIRR, as well as IRF-1, IRF-3, IFN-α and TNF-α [66,67]. In contrast, co-infection induces significantly lower levels of anti-PCV2 and anti-PRRSV IgG antibodies [68,69]. Furthermore, PCV2/PRRSV co-infection plays an immunomodulatory role in the pathogenesis of PCVAD by dramatically decreasing the total and differential leukocyte counts and inducing significantly higher numbers of T(regs) in dendritic cells (DCs) [69,70]. Thereafter, the levels of the inhibitory markers PD-L1 and IL-10 are significantly increased, while the level of the stimulatory markers CD86 is significantly decreased [71]. To date, effective treatment for PRRSV infection is not available, partly because co-infection with PCV2 and PRRSV may interfere with the vaccine. It has been reported that PCV2 vaccination is effective at inducing a neutralizing antibody response and significantly reduces PCV2-associated lesions and viremia in pigs co-infected with PCV2 and PRRSV [72]. However, vaccination with a modified live PRRSV vaccine followed by challenge with PRRSV and PCV2 protected against PRRS but enhanced PCV2 replication and pathogenesis [73]. These results indicate that synergistic effects occur during PRRSV and PCV2 co-infection [62,63], as the clinical signs and lesions in the co-infected pigs are more severe than that of PCV2 or PRRSV singularly infected pigs. Furthermore, mutation rates of PRRSV may increase during PRRSV/PCV2 co-infection.

### 3.3. Porcine Parvovirus

Porcine parvovirus (PPV) infection is the most common and important cause of reproductive failure in swine, which is characterized by embryonic and fetal infection and death [74]. PPV is a virus that normally replicates in the intestines of pigs without causing clinical symptoms. However, when PPV and PCV2 co-infect pigs, obvious clinical symptoms appear.

Coincidental infections of PCV2 and PPV in various combinations have been observed in both pigs and wild boars [11,74,75,76]. Co-infection with PCV2 and PPV in gilts and sows results in litters with mummified and stillborn newborns, as well as seropositive, viable newborns, indicating that antibodies against PCV2 and PPV infections are lacking in dams [77]. Furthermore, experimental inoculation with PCV2 and PPV reproduced lesions similar to those in the field cases of PMWS [78,79,80,81]. Pigs co-infected with PPV and PCV2 appear dull and experience jaundice at approximately 10–12 dpi, and exhibit an enlarged liver and kidneys [78,79,80]. Furthermore, severe macrophage infiltration and granulomatous lesions as well as syncytia and amphophilic inclusion bodies were observed in tissues collected from the viruses of co-infected animals [78,79,80]. These results suggest that the initial viral entry through tonsillar macrophages is followed within 3 days by viremia, and PCV2 and PPV replicate in circulating peripheral monocytes, contributing to cell-associated viremia and viral distribution throughout the lymphoid tissues [81].

It has been reported that co-infection with PCV2 and PPV may promote PCV2 infection by stimulating immune cells and providing target cells for PCV2 replication or by suppressing PCV2 clearance via altering the cytokines production and expression profiles [10,80]. With PCV2 infection, there is a lack of early IFN-γ and TNF-α activation followed by a delayed and low humoral immune response and persistent viremia [82]. In contrast, PPV-infected pigs display activation of IFN-γ and TNF-α, and then initiate an effective immune response to PPV infection [82]. However, pigs infected with PCV2 and PPV display significantly increased expression levels of IL-6, IL-10 and IFN-γ, as well as a strong upregulation of interferon-inducible transmembrane protein 3 (IFITM3) along with several other interferon-stimulated genes (ISGs) [83]. Furthermore, pigs inoculated with both PCV2 and PPV exhibit significantly increased TNF-α compared to pigs inoculated with PCV2 or PPV alone [84]. The levels of TNF-α in the sera are inversely correlated with the body weight of pigs that have been experimentally dually infected with PCV2 and PPV, suggesting that PPV is associated with the excessive production of TNF-α in PCV2-induced PMWS [84]. In a word, PPV and PCV2 play important roles in reproductive failure, which recalls features of the PMWS pathogenesis, and PPV infection provides a better in vivo environment for PCV2 infection.

### 3.4. Classical Swine Fever Virus (CSFV)

Classical swine fever virus (CSFV) is a small, enveloped, plus-strand RNA virus that causes a serious and contagious viral disease in pigs and wild boar worldwide [85].

Although classical swine fever (CSF) has been efficiently controlled by vaccination, increasing clinical evidence has shown co-infection of PCV2 and CSFV in pigs [86]. During co-infection, PCV2 affects CSFV replication in vitro and in vivo. In PK-15 cells, the number of PCV2-CSFV dual-positive cells increases gradually in a PCV2 dose-dependent manner [87]. In CSFV-infected PK15 cells, PCV2 replicates as efficiently as in CSFV-uninfected PK15 cells [87]. Further studies have shown that mitochondrial dysfunction, nuclear factor erythroid 2-related factor 2-mediated oxidative stress response and apoptosis signaling pathways might be specific targets during PCV2-CSFV co-infection [88]. In the field, PCV2 infection can decrease the efficacy of the attenuated CSFV vaccine. PCV2-induced apoptosis might contribute to the impairment of the replication of the attenuated CSFV HCLV strain in co-infected cells [87]. One survey showed that all of the CSFV vaccine-immunized pigs survived and most pigs showed no fever or clinical syndromes; however, pigs co-inoculated with attenuated CSFV and PCV2 showed transient fever, viremia, and viral shedding in the saliva and feces after being challenged with wild-type CSFV [89]. Moreover, when challenged with wild-type CSFV, the number of IgM^+^, CD4^+^CD8^−^CD25^+^, CD4^+^CD8^+^CD25^+^, and CD4^−^CD8^+^CD25^+^ lymphocytes and the level of CSFV-specific neutralizing antibodies were significantly lower in the pigs co-inoculated with PCV2 and the attenuated CSFV compared with that of pigs vaccinated with the attenuated CSFV alone [89]. In addition, the proliferation of peripheral blood mononuclear cells (PBMCs) specifically induced by CSFV was inhibited by PCV2 [89]. The infection and replication level of an attenuated CSFV in alveolar macrophages (AMs) were reduced in a PCV2 dose-dependent manner [90]. These results indicate that PCV2 plays a dominant role during PCV2 and CSFV co-infection. PCV2 may enhance wild-type CSFV infection by inhibiting host immune response, whereas PCV2 could decrease the efficacy of the CSFV vaccine, which should be considered in CSFV vaccination, especially during PCVD.

### 3.5. Swine Influenza Virus (SwIV)

Swine influenza virus (SwIV) is any strain of the influenza family of viruses that is endemic in pigs, including influenza C and the subtypes of influenza A, known as H1N1, H1N2, H2N1, H3N1, H3N2, and H2N3 [91,92].

PRDC is an economically significant problem characterized by slow growth, poor food utilization, lethargy, anorexia, fever, cough, and dyspnea in pigs 16 to 22 weeks old [91,93,94]. Many reports have demonstrated that PCV2 and SwIV play roles in PRDC [10,15,93,94,95,96,97]. Of the 636 SwIV-positive cases collected at the Minnesota Veterinary Diagnostic Laboratory (MVDL) between January 2000 and June 2001, SwIV alone was found in 89 (3.1%) samples; co-infection of PCV2 and SwIV was recorded in 54 (1.9%) samples [98]. A cross-sectional serological study conducted in Bhutan showed that antibodies to the SwIV subtype H1N1 were detected in 49% of the pigs in government farms and in 8% of the village backyard pigs, while these percentages were 73% and 37%, respectively, for PCV2, indicating that PCV2 and SwIV co-infections are also prevalent in pigs [96]. Meiners and colleagues found that SwIV was detected in 53.8% of the samples from weaner pigs with a history of respiratory disease and in 10.6% of sows [92]. The predominant endemic strain was H1N1 (60.5%), whereas subtypes H1N2 (14.0%), H3N2 (14.0%) and human pandemic H1N1 virus or reassortants (11.5%) were detected less frequently [92]. It was found that 3-week-old PCV2-positive pigs were more likely to have SwIV infections and that systemic disease was greater in 16-week-old PCV2-positive pigs than in their PCV2-negative counterparts, indicating that co-infection with PCV2 and SwIV has the greatest effect in the early to late nursery phase [15]. In contrast, co-infection with SwIV did not increase the number of PCV2 genomic copies in the serum or the target tissues, the microscopic lesions associated with PCV2 in lungs or lymph nodes, or the antibody titer against PCV2 in the PCV2-SwIV-infected groups [93]. These results demonstrated that SwIV did not affect PCV2 replication in the co-infected pigs, but PCV2 infection increases SwIV-related clinical disease.

### 3.6. Pseudorabies Virus (PRV)

Pseudorabies virus (Suid herpesvirus 1 or PRV) is a member of the genus *Varicellovirus*, and the family *Herpesviridae* [99]. Although PRV infection has been successfully controlled by different measures in several countries, PRV remains one of the most important pathogens in swine and boar worldwide [99,100]. PRV targets the mucosal epithelium of the pig respiratory and nervous system tissue, causing central nervous system infection and respiratory disease [101].

It was suggested that PCV2 and PRV co-infection is another factor of PRDC. A survey in China reported that 12 samples (18.75%) were positive for co-infection with PRV and PCV-2 among 64 tissues samples, indicating that the co-infection of PCV2 and PRV is also prevalent in the field [102]. However, to date, only a small number of studies have focused on the pathogenesis of the PCV2 and PRV co-infection.

PCV2 infection can downregulate immune cell function during recall antigen responses, and induce IL-10 secretion by monocytic cells, leading to effective repression of IL-12 in PBMCs [103,104]. PRV-induced IFN-α secretion, IL-4 and IL-12p40 upregulation, and IFN-γ expression are also inhibited by PCV2 infection [103,105]. Moreover, PRV was repressed by subsequent PCV2 infection [104]. The ability of PCV2 to hamper the development of immune responses may contribute to the Th1 suppressed responses, immune suppression and co-infections [104]. The PCV2 rep gene and the origin of replication (Ori-rep sequence) can impair the protective cellular immune response induced by DNA vaccination against PRV [106]. These results indicate that the immunosuppressive properties of PCV2 may suppress the host immune response to PRV, resulting in severe PRV infection or vaccination failure against PRV.

### 3.7. Porcine Epidemic Diarrhea Virus (PEDV)

Porcine epidemic diarrhea virus (PEDV) is a member of the genus *Coronavirus*, family *Coronaviridae*, and order *Nidovirales*, and it causes a highly contagious enteric infection in swine with clinical signs including anorexia, vomiting, diarrhea, and dehydration [107,108].

PCV2 is easily detectable in pigs naturally infected with PEDV [108,109]. A survey demonstrated that PCV2 was detected in 32 (29.9%) of 107 small intestinal samples from pigs naturally infected with PEDV [108]. PCV2-positive cells in the jejunum and ileum were found distributed throughout the lamina propria in the small intestine [108]. Furthermore, PCV2 infection can markedly affect the clinical course of PEDV disease [107]. In neonatal piglets, severe anorexia, vomiting and diarrhea were seen in PEDV/PCV2 co-infected piglets within 12 h post inoculation (hpi), while these signs only appeared in piglets infected with PEDV alone at 12 hpi or later [107]. The mean villous height and crypt depth (VH:CD) ratio in sows co-infected with PEDV and PCV2 was significantly decreased than that of sows infected with PEDV alone at 36, 48, and 72 hpi [107]. Moreover, levels of PEDV genomic RNA in PEDV and PCV2 co-infected sows were significantly higher than those in PEDV positive and PCV2 negative sows at 24 hpi, whereas PEDV genomic RNA levels in sows co-infected with PEDV and PCV2 were significantly decreased compared to those of sows infected with PEDV alone at 36, 48, 60 and 72 hpi, possible reason is that PEDV replicated rapidly and destroyed the villous enterocytes during the early infection, resulting in a significant decrease of available cells for PEDV replication in later stages of the infection [107]. These results indicated that co-infection with PCV2 and PEDV resulted in more severe clinical symptoms compared to animals infected with PEDV alone, and PCV2 may enhance PEDV-induced disease and lesions.

### 3.8. Torque Teno Sus Virus (TTSuV)

Torque teno sus virus (TTSuV) belongs to the *Anelloviridae* family, and contains a single-stranded circular DNA genome [110]. To date, it is known that two different TTVs, including Torque teno sus virus 1 (TTSuV1) and TTSuV2 (or TTSuVk2), can infect swine and wild boar [110,111]. TTSuV1 is subgrouped into the species TTSuV1a and TTSuV1b in the genus *Iotatorquevirus*, while TTSuV2 belongs to the genus *Kappatorquevirus* and is divided into the species TSuVk2a and TTSuVk2b [110,111].

TTSuV can infect both healthy and sick pigs. Although several studies have been performed to determine the importance of the virus in infectious diseases, the association between TTSuV and PMWS has been controversial among the different studies [111,112,113,114]. Wasting pigs have a significantly higher load of TTSuV1 and TTSuV2 [113].The percentage of viremic pigs has increased progressively over time, with the highest prevalence in animals approximately 15-week-old [110,112]. The highest TTSuV1 DNA load was found in bone marrow, lung, and liver tissue, while TTSuV2 showed higher loads in bone marrow, mediastinal lymph nodes and liver tissue [115]. Tissues from PCV2-affected pigs showed higher TTSuV2 loads than the tissues of the healthy group [115]. Vlasakova and colleagues found that the rate of co-infection of PCV2 and TTSuV1 was 54.5% in pigs suffering from PMWS [116]. TTSuV1 infection alone stimulates B cell hyperplasia, providing more target cells for PCV2 replication, while both TTSuV2 and PCV2 had a positive impact on macrophage infiltration [113]. These results suggest that the infection dynamics of TTSuV1 and TTSuV2 are different in PCV2 co-infected PMWS pigs than in their uninfected counterparts [110].

In contrast, other groups found that the prevalence of TTSuV1 infection increases with age [112]; however, TTSuVs infection is likely not associated with PCVAD and PMWS [111,112,117,118]. Therefore, whether TTSuVs are associated with PCV2 infection and PMWS needs further investigation.

### 3.9. Co-Infection with Multiple Viruses

Alongside single and dual infections, multiple (including triple or more viruses) infections are also prevalent among pigs. Vlasakova and colleagues evaluated the prevalence of co-infections in five pig groups, including sick suckling pigs, sick post-weaning pigs, sick fattening pigs, and healthy fattening pigs vaccinated with or without PCV2-vaccine [116]. The results demonstrated that sick postweaning pigs and fattening pigs with PMWS were co-infected with multiple viruses [116]. Another survey conducted by Zeng et al. showed that 5 clinical samples were co-infected with PPV, PCV2, PRRSV, and CSFV and the positive rate was 4.8% (5/103) [60]. It was found that PCV2 co-infected with PRRSV enforced clinical signs of PMWS, whereas the influence of other viral co-infections is not clear [116]. Excitingly, vaccination against PCV2 significantly protected pigs against multiple viral infections, except for TTSuVs infection, suggesting that vaccination against PCV2 decreases the risk of PMWS and reduces susceptibility of pigs to the other viral pathogens [116]. These data indicate that PCV2 plays an important role in the co-infection of dual or multiple pathogens in vivo.

## 4. Conclusions and Future Perspectives

Many groups believe that PCV2 alone is not enough to induce PCVD/PCVAD [3,19,20,80]. However, PCV2 targets lymphoid tissues and strongly impacts T-cell selection processes in the thymus [8,119], resulting in an obvious lymphoid depletion and immunosuppression in the pig. Consequently, PCV2 infection results in an increased susceptibility to opportunistic infections of viruses and bacteria. Obviously, co-infections of PCV2 with other viruses may increase pathogenicity in pigs, resulting in more severe clinical symptoms. In addition to dual infections with PCV2 and other viruses, multiple infections are often detected in the field [120]. Moreover, many lines of evidence have shown that bacterial infection, vaccination failure, stress or crowding together with PCV2 can also lead to PCVD/PCVAD [5,14,15,16,17,18,19,20]. In addition, some PCV2-infected pigs can develop severe diseases; however, PCV2 also evokes a subclinical infection in pigs without any obvious symptoms in many cases [121]. Therefore, PCV2 increases clinical signs of PRRSV, CSFV, SwIV, PRV or PEDV infections, and meanwhile, secondary infection (such as PPV infection) and worse physical/growth condition can also provide a better in vivo environment for PCV2 infection. Thus, more research is needed to improve our understanding of the interactions between different swine viruses and bacteria during co-infection with PCV2 in pigs, including how they interact with the host immune response and how they affect the efficacy of vaccination. These studies could lead to important breakthroughs in the understanding of PCVD/PCVAD and in the development of new strategies to control the disease.
